# Prognostic value of CD169-positive macrophages in various tumors: a meta-analysis

**DOI:** 10.1080/21655979.2021.1985857

**Published:** 2021-10-22

**Authors:** Weihao Kong, Meng Wei, Rongqiang Liu, Jianlin Zhang, Xingyu Wang

**Affiliations:** a Department of Emergency Surgery, Department of Emergency Medicine, The First Affiliated Hospital of Anhui Medical University, Hefei, China; b Department of General Surgery, The First Affiliated Hospital of Anhui Medical University, Hefei, China; c Department of Hepatobiliary Surgery, The First Affiliated Hospital of Guangzhou Medical University, Guangzhou, Guangdong, China

**Keywords:** CD169, macrophages, prognosis, meta-analysis

## Abstract

The study aimed to evaluate the prognostic value of CD169 expression in tumor-infiltrating macrophages from regional lymph nodes (RLN) in various tumors. In order to identify eligible articles, PubMed, EMBASE, Web of Science, and Cochrane Library were used to conduct a systematic search. Pooled hazard ratios (HRs) or odds ratios (ORs) with corresponding 95% confidence intervals (CIs) were adopted to assess the relationship between CD169 expression and overall survival (OS) and clinicopathological characteristics. Ten studies, including eleven cohorts with 1699 patients, were enrolled. We found that high CD169^+^ expression in tumor-infiltrating macrophages from RLN was associated with a favorable OS (HR = 0.56, 95%CI: 0.39–0.79, P = 0.001). Subgroup analysis showed that high CD169+ expression had more predictive power in digestive system tumors (HR = 0.52, 95%CI: 0.42–0.67, <0.001). In addition, high CD169 expression was significantly linked with lymph node metastasis (OR = 0.66, 95%CI: 0.47–0.94, P = 0.020) and TNM stage (OR = 0.62, 95%CI: 0.48–0.80, P < 0.001). High CD169 expression in tumor-infiltrating macrophages from RLN was correlated with favorable survival outcome in patients with malignancies. CD169 may be a novel and effective prognostic marker, especially for digestive system tumors.

## GRAPHICAL ABSTRACT

**Figure:**
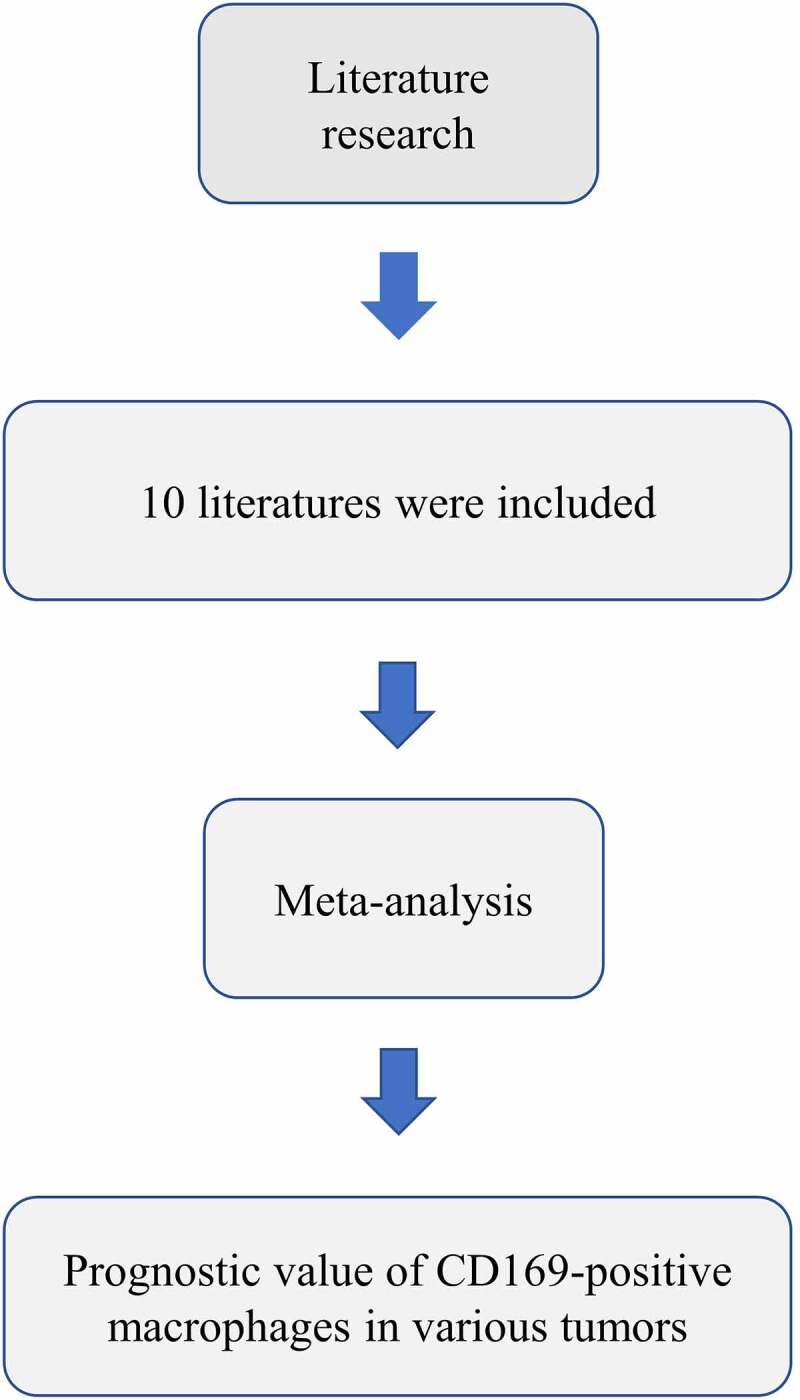

## Introduction

The incidence and mortality of cancer is rapidly growing because of the aging and growth of the population as well as socio-economic development [1,2]. According to the statistics, 18.1 million newly diagnosed cancer cases and 9.6 million cancer-related deaths occurred in 2018 [1]. Despite advanced drug treatments and surgical methods, the therapeutic response differs significantly in patients, and the overall prognosis of most cancers is still not very satisfactory [3]. The complex molecular mechanisms are the features of cancer cells. Therefore, it is imperative for us to explore applicable predictive biomarkers so that we enable to accurately evaluate accurately the therapeutic effect and prognosis of patients with malignancies.

Macrophages, an indispensable component of the innate immune system, are spread across the body [4]. Plenty of tumor-associated macrophages are detected in tumors, and them contribute to the major inflammatory infiltrate in various malignancies [5,6]. Macrophages have both pro- and anti-tumorigenic functions in different local tumor environmental conditions. The regional lymph nodes(RLN) is one of the first major components of the immune system that comes into contact with tumor cells or tumor cell products, and plays an important role in generating tumor-directed immune responses [7]. The macrophages located in RLN also play an important role in tumor immunology through endocytosing the fragmented dead tumor cells of the sinus area of the lymph nodes [7–10]. These findings suggest that regional lymph nodes or tumor-infiltrating macrophages represent a pivotal component in various tumor immunology.

CD169 (also known as Siglec-1) is a surface marker of macrophages and belongs to the sialic-acid-binding immunoglobulin-like lectin family, which can mediate cell-cell interactions via glycan recognition [11,12]. Nowadays, researchers recommended that CD169 expression in tumor-infiltrating macrophage from RLN served as a promising prognostic marker of multiple cancers [13–19]. However, their conclusions were contradictory. The prognostic value of CD169 expression in tumor-infiltrating macrophages from RLN was still unclear. Therefore, we conducted this meta-analysis to clarify the prognostic power of CD169 in cancers.

## Materials and methods

### Search strategy

A systematic search for eligible articles was conducted through PubMed, Web of Science, EMBASE, and Cochrane Library database. The search deadline was up to December 2020. The following keywords were used: ‘CD169’ OR ‘sialoadhesin’ OR ‘Siglec-1’ AND ‘cancer’ OR ‘carcinoma’ OR ‘neoplasm’ OR ‘tumor’ AND ‘prognosis’ OR ‘prognostic’ OR ‘survival’ OR ‘outcome’ AND ‘Macrophage’. Additionally, the reference lists of the included studies were explored for potentially relevant articles.

### Study selection

The searching tasks were carried out independently by three authors (Kong WH, Wei and Liu RQ). Selection criteria employed in this meta-analysis to incorporate eligible studies were as follows: 1) Original English publication, 2) investigated the association between CD169 and single or multiple malignancies, 3) focusing on the CD169 and clinical outcomes.

### Data extraction and quality assessment

Data extraction was performed by three reviewers (Kong WH, Wei M and Liu RQ) independently. The fourth reviewer (Zhang JL) would be involved in the discussion when any discrepancies were met. Extracted data and information included as follows: 1) The first author and the year of publication, 2) Article nationality, 3) Type of cancer, 4) The case number of included studies, 5) Median age of patients, 6) Test method of CD169 expression, 7) Definition of high or low CD169+ macrophages, 8) Location of the specimen, 9) Survival results, 10) Follow-up months. The quality of each included study was evaluated using the Newcastle-Ottawa scale (NOSs) [20,21]. NOS score from 0–4 was defined as low quality, 5–6 as medium quality, and 7–9 as high quality.

### Statistical analysis

The pooled hazard ratios (HRs)or odds ratios (ORs) with the 95% confidence intervals (CIs) were calculated to illustrate the relationship between CD169 expression and survival outcome and clinicopathological characteristics. Cochran’s Q test and I^2^ statistics were performed to evaluate heterogeneity [22,23]. Heterogeneity was perceived as significant when I^2^ > 50% or p < 0.1, which determined the adoption of random effect models. Otherwise, fixed effect models were chosen. Moreover, the stability of the merged result was assessed by sensitivity analysis. Begg’s and Egger’s tests were utilized to detect the publication bias [24]. STATA 12.0 (StataCorp, College Station, TX, USA) was employed to conduct statistical analyses. P less than 0.05 was considered as statistical difference.

## Results

### Brief introduction

CD169 macrophages have been reported to play a pivotal role in anti-tumor immunity, but its prognostic value remains controversy. Therefore, a meta-analysis evaluating the prognostic value value of CD169 expression in tumor-infiltrating macrophages from regional lymph nodes was conducted. We retrieved articles about the relationship between CD169 expression and prognostic value in multiple tumors from the database. Through meta-analysis, we found that the high CD169 expression was significantly related to favorable prognosis, lymph node metastasis, and TNM stage.

### Literature search and study characteristics

**Figure 1.**
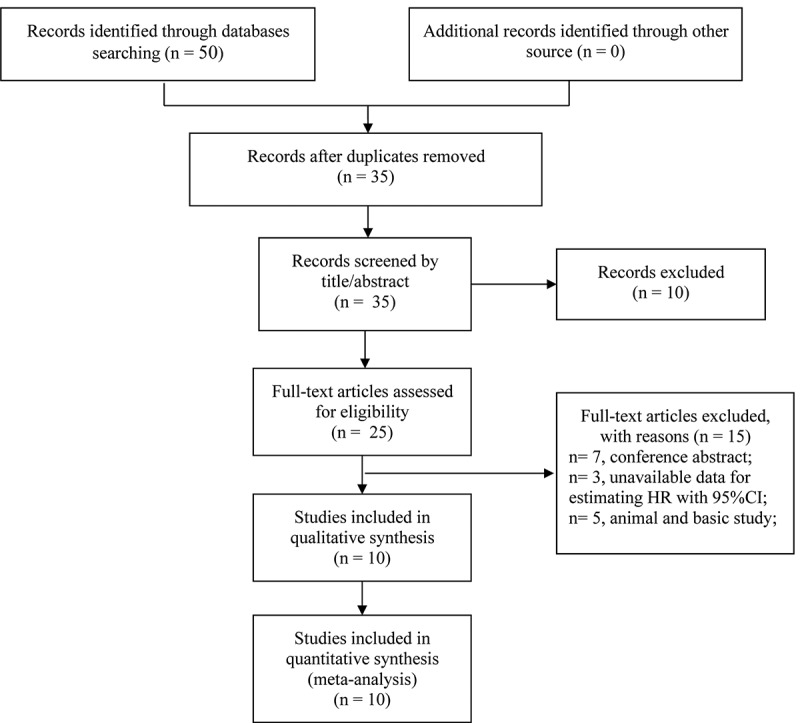
Flow diagram of the study selection process

Literature selection flow was shown in Figure 1 in accordance with the PRISMA statement [25]. A total of 50 studies were initially identified through the search strategy described above. However, 15 studies were removed for duplication, and 25 studies were excluded based on the titles, abstracts, and full texts. Eventually, ten studies with 1699 patients were incorporated in the present meta-analysis. Table 1. summarizes the baselines characteristics of selected ten studies. The malignant neoplasm contained bladder cancer, esophageal cancer, hepatocellular carcinoma, gastric cancer, colorectal carcinoma, endometrial carcinoma, melanoma, breast cancer, prostate cancer, and bladder urothelial carcinoma. Surgically resected tissues were collected to detect the CD169 expression in all studies. As for survival results, overall survival (OS) was reported in nine studies; one research displayed the OS and relapse-free survival (RFS). All the included studies were retrospective. Follow-up time among studies varied from 82 months to 250 months in ten studies. The quality assessment of the included studies was presented in Table 2.

**Table 1.** Baseline characteristics of studies included in the meta-analysis

Study	Origin	Cancer Type	Cases (low/high)	Gender (male/female)	Tumor stage	Median age (range)	Test method	Definition of high or low CD169+ macrophages	Location	Survival results	Maximum months of follow-up
Asano 2018	Japan	Bladder cancer	44 (26/18)	35/9	TNM (I–IV)	70 (49–85)	IHC	Mean number of cells and mean intensity	Regional lymph nodes	OS^MA^	133
Hiroto 2018	Japan	Esophageal cancer	182 (101/81)	160/22	TNM (I–IV)	NA	IHC	Mean densities	Regional lymph nodes	OS^MA^	140
Li 1**#** 2017	China	Hepatocellular carcinoma	188 (94/94)	156/29	TNM (I–III)	50 (13–76)	IHC	Mean densities	Intra-tumor	OS^MA^	120
Li 2**#** 2017	China	Gastric cancer	132 (66/66)	95/37	TNM (I–IV)	69 (28–78)	IHC	Mean densities	Intra-tumor	OS^MA^	120
Ohnishi 2013	Japan	Colorectal carcinoma	83 (45/38)	48/35	TNM (I–IV)	64 (29–90)	IHC	Mean number of cells	Regional lymph nodes	OS^UA^	100
Ohnishi 2016	Japan	Endometrial carcinoma	79 (39/40)	NA	FIGO (I–IV)	59 (30–74)	IHC	Mean number of cells	Regional lymph nodes	OS^UA^	120
Saito 2015	Japan	Melanoma	84	36/48	Stage (0–4)	69 (34–91)	IHC	Mean number of cells	Regional lymph nodes	OS^MA^	100
Shiota 2016	Japan	Breast Cancer	146	73/73	Stage (1–3)	56 (NA)	IHC	Mean number of cells	Regional lymph nodes	OS^MA,^ RFS^MA^	159
Strömvall 2017	Sweden	Prostate cancer	109 (27/82)	NA	Gleason (6–9)	NA	IHC	Mean densities	Regional lymph nodes	OS^MA^	250
Wang 2015	China	Bladder carcinoma	302 (151/151)	262/40	TNM (0-IV)	60 (15–90)	IHC	Mean number of cells and mean intensity	Intra-tumor	OS^MA^	82
Zhang 2016	China	Hepatocellular carcinoma	328 (164/164)	292/36	TNM (I–III)	48 (20–78)	IHC	Mean densities	Intra-tumor	OS^MA^	96

This means that these two different studies are from the same article.

Abbreviations: NA, not available; IHC, immunohistochemistery; OS, overall survival; RFS, recurrence-free survival; MA, multivariate analysis; UA, univariate analysis.

**Table 2.** Newcastle -Ottawa Quality Assessment Scale

Author	Year	Selection	Comparability	Outcome	Total
Asano et al.	2018	☆☆	☆☆	☆☆☆	7
Hiroto et al	2018	☆☆	☆☆	☆☆	6
Li et al. 1	2017	☆☆	☆☆	☆☆	6
Li et al. 2	2017	☆☆	☆☆	☆☆	6
Ohnishi et al.	2013	☆	☆☆	☆☆☆	6
Ohnishi et al.	2016	☆☆	☆☆	☆☆☆	7
Saito et al.	2015	☆☆	☆☆	☆☆☆	7
Shiota et al.	2016	☆☆	☆☆	☆☆	6
Stromvall et al.	2017	☆☆	☆☆	☆☆☆	7
Wang et al.	2015	☆	☆☆	☆☆☆	6
Zhang et al.	2016	☆☆	☆☆	☆☆☆	7

### Association between CD169 expression and OS

**Figure 2.**
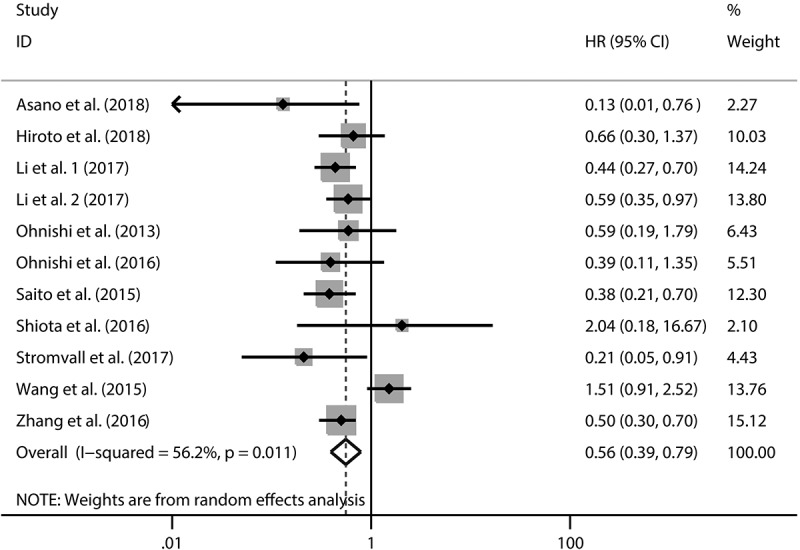
Forest plot of the overall survival analysis

Ten studies reported the association between CD169 expression and OS. we found a significant relationship between high CD169 expression and longer OS in cancers (HR: 0.56, 95%CI: 0.39–0.79, P = 0.001) (Figure 2). Additionally, the heterogeneity among the studies (I2 = 56.2%, P = 0.011) was significant. To explore the source of heterogeneity, subgroup analysis for OS was performed (Table 3). The results were displayed in Table 3. We found that the sources of heterogeneity included country, tumor type, sample size, definition of high or low, macrophages location, analysis type and NOS score. In addition, we also observed that high CD169+ expression had more predictive power in digestive system tumors (HR = 0.52, 95%CI: 0.42–0.67, <0.001).

**Table 3.** Subgroup analysis for overall survival

Stratified analysis	No. of cohorts	Pooled HR (95% CI)	*P*-value	Heterogeneity		
				*I*^2^ (%)	*P*-value	Model
Country						
Janpan	6	0.48(0.32, 0.71)	<0.001	0	0.482	Random
China	4	0.66(0.39, 1.12)	0.124	79.9	0.002	Random
Sweden	1	0.21(0.05, 0.90)	0.035	-	-	Random
Tumor type						
Digestive system	5	0.52(0.42, 0.67)	<0.001	0	0.877	Random
Urinary system	3	0.42(0.08, 2.34)	0.324	80.5	0.006	Random
Others	3	0.42(0.25, 0.71)	0.001	0	0.370	Random
Sample size						
≤ 100	4	0.39(0.24, 0.63)	<0.001	0	0.678	Random
>100	7	0.63(0.41, 0.98)	0.039	67.0	0.006	Random
Definition of high or low						
Mean number of cells	4	0.45(0.28, 0.72)	0.001	0	0.515	Random
Mean densities	5	0.50(0.39, 0.65)	<0.001	0	0.629	Random
Both	2	0.56(0.05, 5.95)	0.632	78.6	0.031	Random
Macrophages location						
Regional lymph nodes	7	0.45(0.31, 0.66)	<0.001	0	0.471	Random
Intra-tumor	4	0.66(0.39, 1.12)	0.124	79.9	0.002	Random
Analysis type						
Univariate	2	0.49(0.21, 1.13)	0.095	0	0.630	Random
Multivariate	9	0.56(0.38, 0.84)	0.004	64.3	0.004	Random
NOS score						
≤6	6	0.73(0.45, 1.18)	0.198	64.4	0.015	Random
>6	5	0.42(0.31, 0.58)	<0.001	0	0.597	Random

### Association between CD169 expression and clinicalpathological parameters

We summarized the data to assess the relationship between CD169 expression and clinicopathological characteristics (Table 4). The combined results indicated that high CD169 expression was obviously related with TNM stage (III–IV vs I–II) (OR = 0.62, 95% CI: 0.48, 0.80, P < 0.001) and Lymph node metastasis (yes vs no)(OR = 0.66, 95% CI: 0.47–0.94; P = 0.020). However, the correlation was not observed in vascular invasion and histological grade. We believed that high CD169 expression may play an important role in preventing tumor invasion and lymph node metastasis

**Table 4.** Association between CD169+ macrophages and clinicopathological features

Clinicopathological parameter	No. of cohorts	Model	OR (95% CI)	*P*-value	Heterogeneity test	
					*I*^2^(%)	*P*-value
Lymph node metastasis (yes vs no)	6	Fixed	0.66(0.47,0.94)	**0.020**	0.8	0.411
TNM stage (III–IV vs I–II)	7	Fixed	0.62(0.48,0.80)	**<0.001**	0	0.839
Vascular invasion (yes vs no)	4	Fixed	0.79(0.52,1.20)	0.271	0	0.999
Histological grade (III vs I–II)	4	Fixed	1.26(0.90,1.76)	0.177	41.0	0.165

### Sensitivity analysis

**Figure 3.**
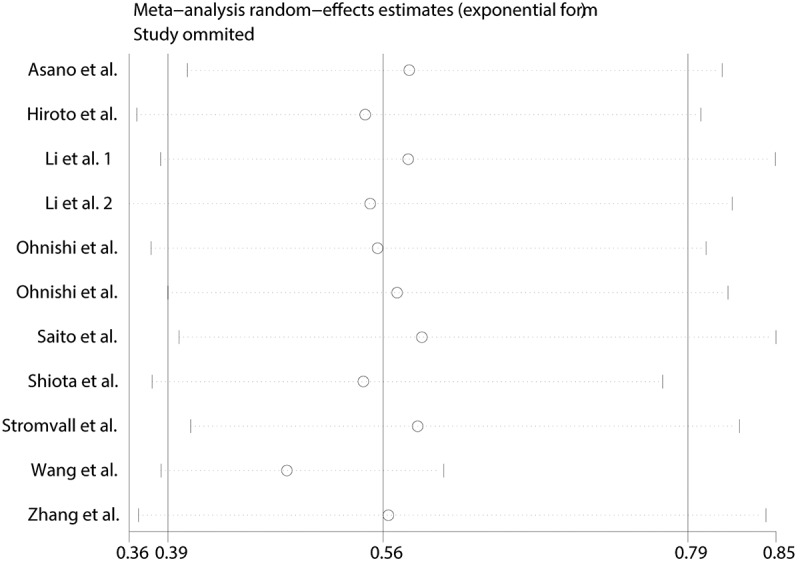
Sensitivity analysis of overall survival in this meta-analysis

Sensitivity analysis was employed to assess the influence of each individual study on the pooled HR. The results did not vary substantially with the exclusion of each research, which demonstrated stability and reliability of our results (Figure 3).

### Publication bias

**Figure 4.**
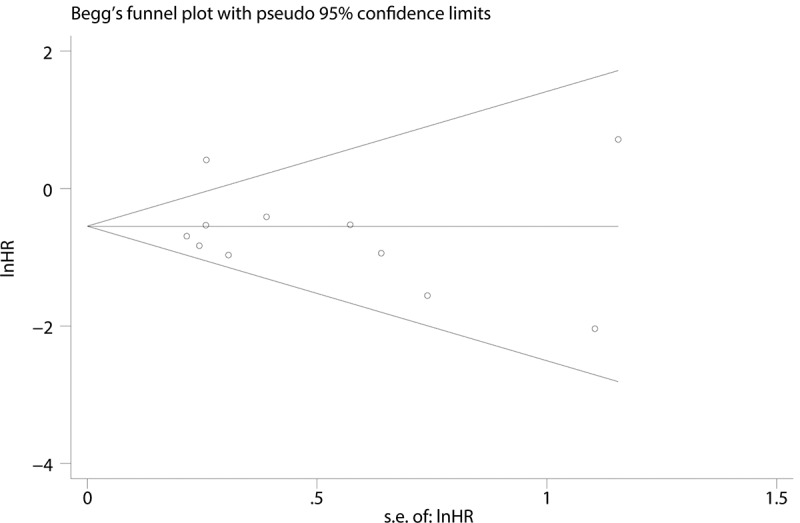
Begg’s funnel plot with pseudo 95% CI of the publication bias for overall survival

The publication bias was detected by Begg’s tests and Egger’s tests. P-values for the Begg’s and Egger’s tests for OS were 0.876 and 0.587, respectively. P values were greater than 0.05, indicating no publication bias (Figure 4).

## Discussion

Macrophages, which are consisted of diverse subpopulations based on specific markers, have diverse functions, including both pro-tumor and anti-tumor functions [26,27]. Macrophages in various cancers differ in distribution and composition patterns. Sinus macrophages play a pivotal role in anti-tumor immunity by endocytosing dead tumor cells, presenting antigens, and activating tumor antigen-specific lymphocytes [10,28]. CD169, which is a sialic acid receptor expressed on macrophages, is involved in the process of cell-cell interactions and cell-pathogen interactions. Increasing evidences have suggested that CD169+ macrophages play a crucial role in the anti-tumor immune response [28,29].

In the present study, a significant relationship was revealed between the high CD169 expression and OS (HR: 0.56, 95%CI: 0.39–0.79, P = 0.001). Subgroup analysis showed that the abundance of CD169 macrophages had a significant relationship with OS in Japan (HR: 0.48, 95%CI: 0.32–0.71, P < 0.001) and Sweden (HR: 0.21, 95%CI: 0.05–0.90, P = 0.035). We speculated that this may be caused by differences in genes, geography and climate. Moreover, high CD169+ expression had more predictive power in digestive system tumors(HR = 0.52, 95%CI: 0.42–0.67, <0.001). Additionally, CD169 macrophages from regional lymph nodes correlated with OS (HR: 0.45, 95% CI: 0.31–0.66, P < 0.001) rather than that intra-tumor macrophages (HR: 0.66, 95% CI: 0.39–1.12, P = 0.124). It suggested that macrophage’s location should be taken into consideration when using CD169 macrophages as prognostic value. In addition. It revealed that lymph node metastasis (OR: 0.66, 95% CI: 0.47–0.94, P = 0.020) and TNM stage (OR: 0.62, 95% CI: 0.48–0.80, P < 0.001) were associated prominently with high CD 169 macrophages.

Mechanisms underlying the prognostic value of CD169 expression in macrophages may be as follows. Touko Asano et al. revealed that the amount of CD169+ macrophages significantly correlated with the abundance of CD8+ cells and the favorable survival in bladder cancer, suggesting that CD169+ macrophages may play an anti-tumor role by boosting cytotoxic T-cell-mediated anti-tumor immunity [18]. Similarly, positive results between the density of CD169+ macrophages and CD8+ T cell infiltration were discovered in other cancers, including hepatocellular carcinoma, gastric cancer, and colorectal carcinoma [13,30]. Ohnishi and his colleagues identified that sinus macrophages and CD8+ T cells interaction were mediated by CD169–CD43 ligation in a regional lymph node, which may be one of the mechanisms for the proliferation of CD8+ T cells. Additionally, Hiroto Takeya et al. revealed a positive association between higher CD169 expression and density of tumor-infiltrating lymphocytes in esophageal cancer who underwent neoadjuvant chemotherapy, indicating that high CD169 expression plays a crucial role in inducing anti-cancer immune responses [19]. Moreover, activating NK cell-mediated anti-tumor immunity may be one of the mechanisms. Based on Garcia and Coombes’s experiment, Koji Ohnishi and his colleagues demonstrated that CD169+ macrophages activate infiltrating NK cells in the tumor by direct contact with CD57+ NK cells in RLN [15]. Besides, regulatory T cells (Treg) were suppressed by interaction with CD 169 and result in inflammation [31]. Taken together with our meta-analysis, we believed CD169+ macrophages may play a crucial role in anti-tumor immunity.

Several limitations should be taken into consideration. Firstly, all included studies were small retrospective studies. Secondly, the definition of the high CD169 expression varied among different researches. Thirdly, due to the limited included articles, we performed a subgroup analysis of the same systemic tumors. However, due to differ in their ontogeny, management and prognosis, the subgroup analysis may lead to erroneous results. Finally, significant heterogeneity is found in this meta-analysis, and the results should be treated with caution

## Conclusion

High CD169 expression in macrophages from RLN predicted favorable survival outcome in patients with cancers, especially for digestive system tumors. CD169 could be an ideal prognositic marker in tumors. CD169 can be used to determine the prognosis of tumor patients and help clinicians to implement personalized treatment in advance. However, due to unavoidable restrictions, more large-scale, multi-center studies are needed to confirm our findings.

## Data Availability

The data used to support the findings of this research are available from the corresponding author upon request.
